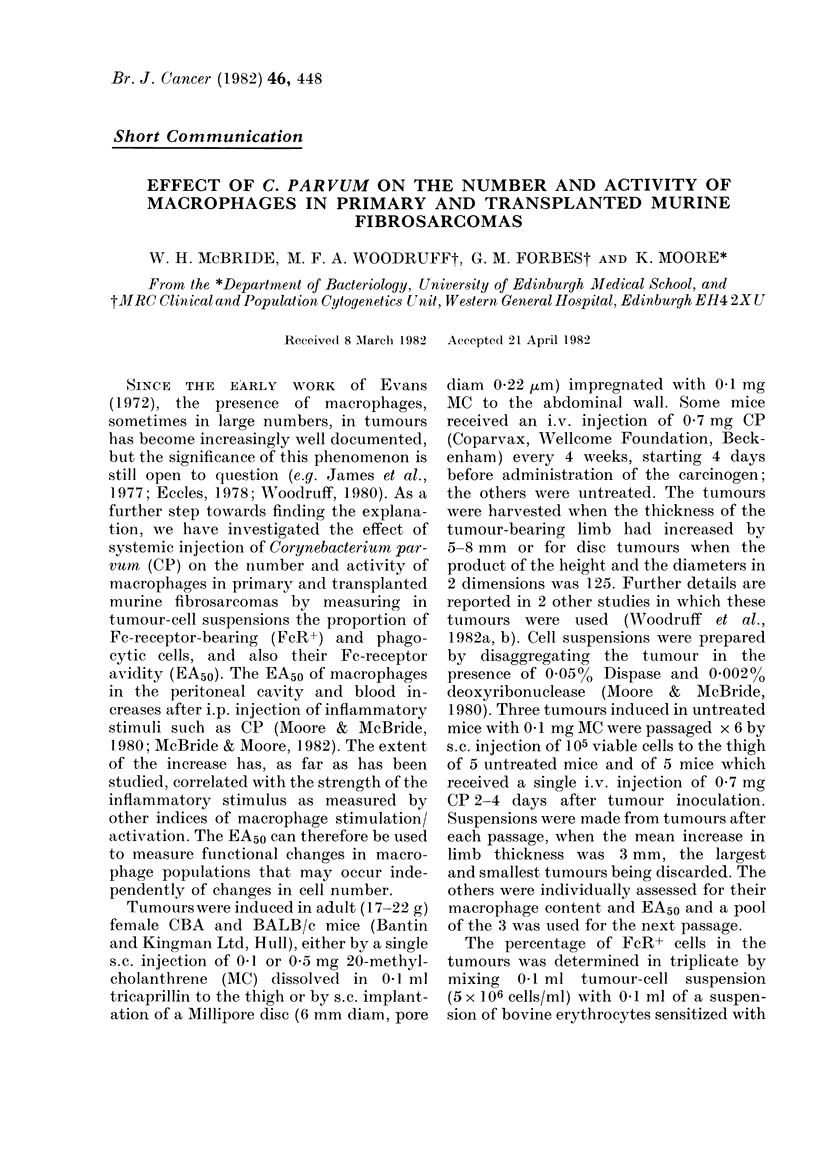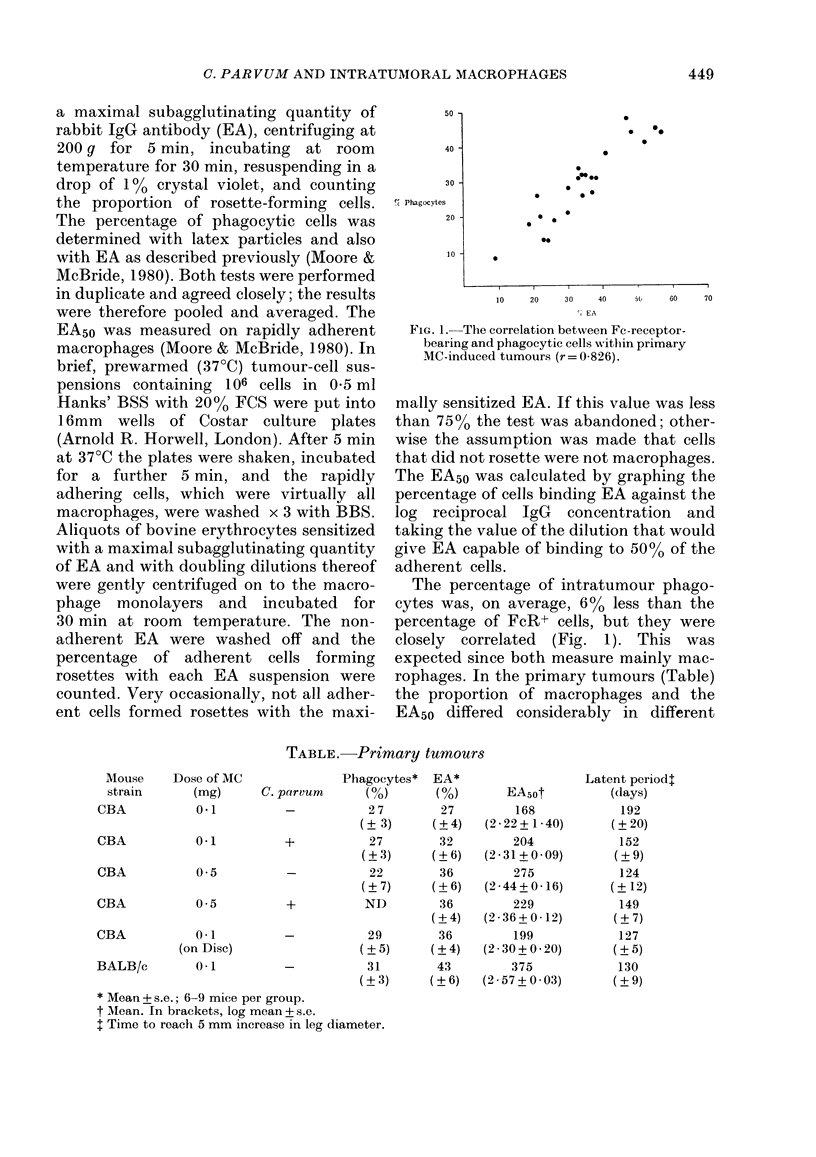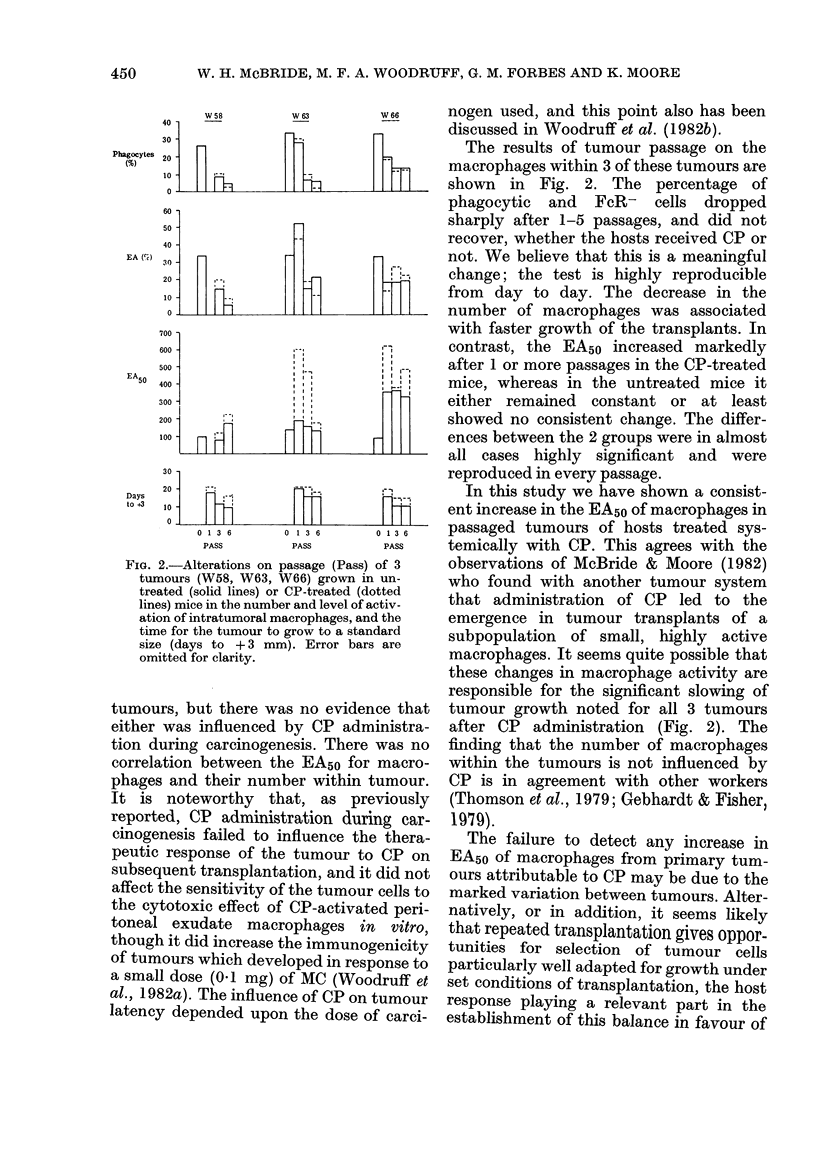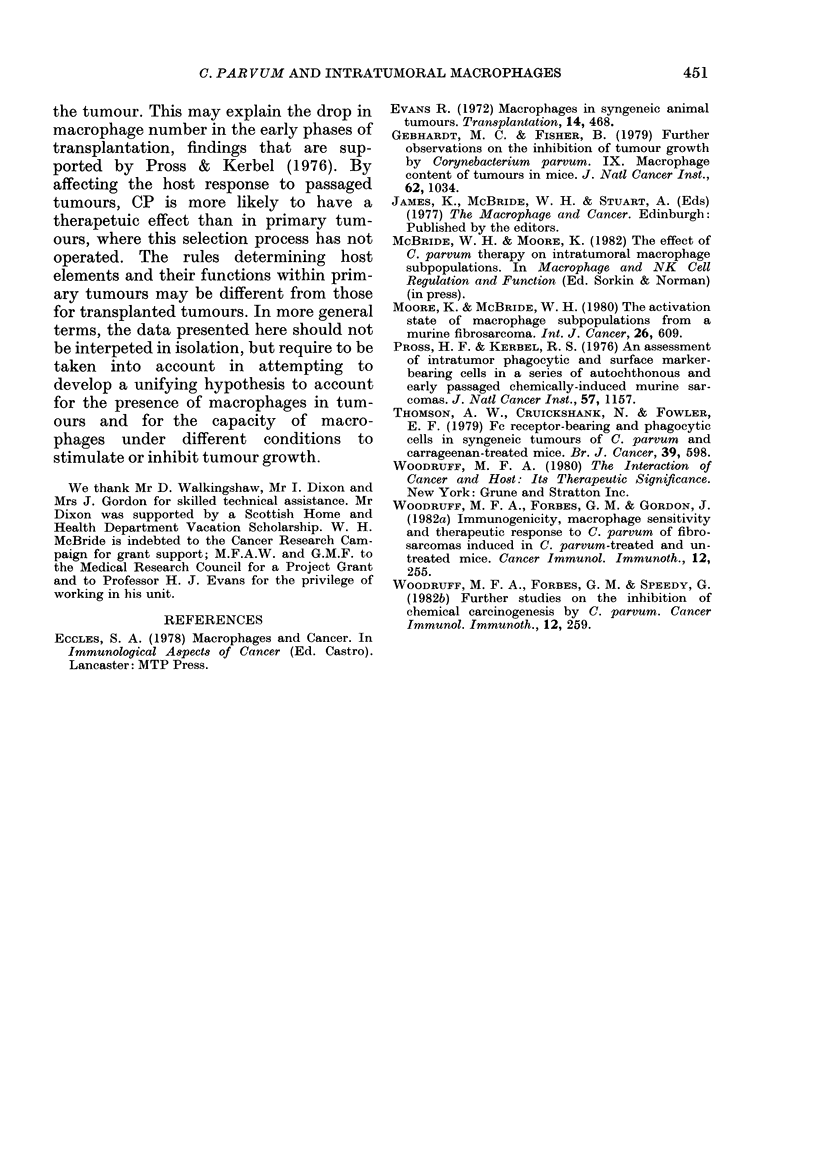# Effect of C. parvum on the number and activity of macrophages in primary and transplanted murine fibrosarcomas.

**DOI:** 10.1038/bjc.1982.224

**Published:** 1982-09

**Authors:** W. H. McBride, M. F. Woodruff, G. M. Forbes, K. Moore


*Br. J. Cancer* (1982) **46**, 448

***Short Communication***

### EFFECT OF *C. PARVUM* ON THE NUMBER AND ACTIVITY OF MACROPHAGES IN PRIMARY AND TRANSPLANTED MURINE FIBROSARCOMAS

W. H. McBRIDE, M. F. A. WOODRUFF†, G. M. FORBES† AND K. MOORE*

*From the *Department of Bacteriology, University of Edinburgh Medical School, and †MRC Clinical and Population Cytogenetics Unit, Western General Hospital, Edinburgh EH4 2XU*

Received 8 March 1982 Accepted 21 April 1982

SINCE THE EARLY WORK of Evans (1972), the presence of macrophages, sometimes in large numbers, in tumours has become increasingly well documented, but the significance of this phenomenon is still open to question (*e.g.* James *et al.*, 1977; Eccles, 1978; Woodruff, 1980). As a further step towards finding the explanation, we have investigated the effect of systemic injection of *Corynebacterium parvum* (CP) on the number and activity of macrophages in primary and transplanted murine fibrosarcomas by measuring in tumour-cell suspensions the proportion of Fc-receptor-bearing ($FcR^+$) and phagocytic cells, and also their Fc-receptor avidity ($EA_{50}$). The $EA_{50}$ of macrophages in the peritoneal cavity and blood increases after i.p. injection of inflammatory stimuli such as CP (Moore & McBride, 1980; McBride & Moore, 1982). The extent of the increase has, as far as has been studied, correlated with the strength of the inflammatory stimulus as measured by other indices of macrophage stimulation/activation. The $EA_{50}$ can therefore be used to measure functional changes in macrophage populations that may occur independently of changes in cell number.

Tumours were induced in adult (17–22 g) female CBA and BALB/c mice (Bantin and Kingman Ltd, Hull), either by a single s.c. injection of 0·1 or 0·5 mg 20-methylcholanthrene (MC) dissolved in 0·1 ml tricaprillin to the thigh or by s.c. implantation of a Millipore disc (6 mm diam, pore diam 0·22 $\mu$m) impregnated with 0·1 mg MC to the abdominal wall. Some mice received an i.v. injection of 0·7 mg CP (Coparvax, Wellcome Foundation, Beckenham) every 4 weeks, starting 4 days before administration of the carcinogen; the others were untreated. The tumours were harvested when the thickness of the tumour-bearing limb had increased by 5–8 mm or for disc tumours when the product of the height and the diameters in 2 dimensions was 125. Further details are reported in 2 other studies in which these tumours were used (Woodruff *et al.*, 1982a, b). Cell suspensions were prepared by disaggregating the tumour in the presence of 0·05% Dispase and 0·002% deoxyribonuclease (Moore & McBride, 1980). Three tumours induced in untreated mice with 0·1 mg MC were passaged $\times$ 6 by s.c. injection of $10^5$ viable cells to the thigh of 5 untreated mice and of 5 mice which received a single i.v. injection of 0·7 mg CP 2–4 days after tumour inoculation. Suspensions were made from tumours after each passage, when the mean increase in limb thickness was 3 mm, the largest and smallest tumours being discarded. The others were individually assessed for their macrophage content and $EA_{50}$ and a pool of the 3 was used for the next passage.

The percentage of $FcR^+$ cells in the tumours was determined in triplicate by mixing 0·1 ml tumour-cell suspension ($5 \times 10^6$ cells/ml) with 0·1 ml of a suspension of bovine erythrocytes sensitized with a maximal subagglutinating quantity of rabbit IgG antibody (EA), centrifuging at 200 *g* for 5 min, incubating at room temperature for 30 min, resuspending in a drop of 1% crystal violet, and counting the proportion of rosette-forming cells. The percentage of phagocytic cells was determined with latex particles and also with EA as described previously (Moore & McBride, 1980). Both tests were performed in duplicate and agreed closely; the results were therefore pooled and averaged. The $EA_{50}$ was measured on rapidly adherent macrophages (Moore & McBride, 1980). In brief, prewarmed (37°C) tumour-cell suspensions containing $10^6$ cells in 0·5 ml Hanks' BSS with 20% FCS were put into 16mm wells of Costar culture plates (Arnold R. Horwell, London). After 5 min at 37°C the plates were shaken, incubated for a further 5 min, and the rapidly adhering cells, which were virtually all macrophages, were washed × 3 with BBS. Aliquots of bovine erythrocytes sensitized with a maximal subagglutinating quantity of EA and with doubling dilutions thereof were gently centrifuged on to the macrophage monolayers and incubated for 30 min at room temperature. The non-adherent EA were washed off and the percentage of adherent cells forming rosettes with each EA suspension were counted. Very occasionally, not all adherent cells formed rosettes with the maximally sensitized EA. If this value was less than 75% the test was abandoned; otherwise the assumption was made that cells that did not rosette were not macrophages. The $EA_{50}$ was calculated by graphing the percentage of cells binding EA against the log reciprocal IgG concentration and taking the value of the dilution that would give EA capable of binding to 50% of the adherent cells.

FIG. 1.—The correlation between Fc-receptor-bearing and phagocytic cells within primary MC-induced tumours ($r = 0·826$).

The percentage of intratumour phagocytes was, on average, 6% less than the percentage of $FcR^+$ cells, but they were closely correlated (Fig. 1). This was expected since both measure mainly macrophages. In the primary tumours (Table) the proportion of macrophages and the $EA_{50}$ differed considerably in different

TABLE.—*Primary tumours*

| Mouse strain | Dose of MC (mg) | *C. parvum* | Phagocytes* (%) | EA* (%) | $EA_{50}$† | Latent period‡ (days) |
|---|---|---|---|---|---|---|
| CBA | 0·1 | – | 27 (± 3) | 27 (±4) | 168 (2·22±1·40) | 192 (±20) |
| CBA | 0·1 | + | 27 (±3) | 32 (±6) | 204 (2·31±0·09) | 152 (±9) |
| CBA | 0·5 | – | 22 (±7) | 36 (±6) | 275 (2·44±0·16) | 124 (±12) |
| CBA | 0·5 | + | ND | 36 (±4) | 229 (2·36±0·12) | 149 (±7) |
| CBA | 0·1 (on Disc) | – | 29 (±5) | 36 (±4) | 199 (2·30±0·20) | 127 (±5) |
| BALB/c | 0·1 | – | 31 (±3) | 43 (±6) | 375 (2·57±0·03) | 130 (±9) |

* Mean±s.e.; 6–9 mice per group.
† Mean. In brackets, log mean±s.e.
‡ Time to reach 5 mm increase in leg diameter.

FIG. 2.—Alterations on passage (Pass) of 3 tumours (W58, W63, W66) grown in untreated (solid lines) or CP-treated (dotted lines) mice in the number and level of activation of intratumoral macrophages, and the time for the tumour to grow to a standard size (days to +3 mm). Error bars are omitted for clarity.

tumours, but there was no evidence that either was influenced by CP administration during carcinogenesis. There was no correlation between the $EA_{50}$ for macrophages and their number within tumour. It is noteworthy that, as previously reported, CP administration during carcinogenesis failed to influence the therapeutic response of the tumour to CP on subsequent transplantation, and it did not affect the sensitivity of the tumour cells to the cytotoxic effect of CP-activated peritoneal exudate macrophages *in vitro*, though it did increase the immunogenicity of tumours which developed in response to a small dose (0·1 mg) of MC (Woodruff *et al.*, 1982*a*). The influence of CP on tumour latency depended upon the dose of carcinogen used, and this point also has been discussed in Woodruff *et al.* (1982*b*).

The results of tumour passage on the macrophages within 3 of these tumours are shown in Fig. 2. The percentage of phagocytic and $FcR^-$ cells dropped sharply after 1–5 passages, and did not recover, whether the hosts received CP or not. We believe that this is a meaningful change; the test is highly reproducible from day to day. The decrease in the number of macrophages was associated with faster growth of the transplants. In contrast, the $EA_{50}$ increased markedly after 1 or more passages in the CP-treated mice, whereas in the untreated mice it either remained constant or at least showed no consistent change. The differences between the 2 groups were in almost all cases highly significant and were reproduced in every passage.

In this study we have shown a consistent increase in the $EA_{50}$ of macrophages in passaged tumours of hosts treated systemically with CP. This agrees with the observations of McBride & Moore (1982) who found with another tumour system that administration of CP led to the emergence in tumour transplants of a subpopulation of small, highly active macrophages. It seems quite possible that these changes in macrophage activity are responsible for the significant slowing of tumour growth noted for all 3 tumours after CP administration (Fig. 2). The finding that the number of macrophages within the tumours is not influenced by CP is in agreement with other workers (Thomson *et al.*, 1979; Gebhardt & Fisher, 1979).

The failure to detect any increase in $EA_{50}$ of macrophages from primary tumours attributable to CP may be due to the marked variation between tumours. Alternatively, or in addition, it seems likely that repeated transplantation gives opportunities for selection of tumour cells particularly well adapted for growth under set conditions of transplantation, the host response playing a relevant part in the establishment of this balance in favour of the tumour. This may explain the drop in macrophage number in the early phases of transplantation, findings that are supported by Pross & Kerbel (1976). By affecting the host response to passaged tumours, CP is more likely to have a therapetuic effect than in primary tumours, where this selection process has not operated. The rules determining host elements and their functions within primary tumours may be different from those for transplanted tumours. In more general terms, the data presented here should not be interpeted in isolation, but require to be taken into account in attempting to develop a unifying hypothesis to account for the presence of macrophages in tumours and for the capacity of macrophages under different conditions to stimulate or inhibit tumour growth.

We thank Mr D. Walkingshaw, Mr I. Dixon and Mrs J. Gordon for skilled technical assistance. Mr Dixon was supported by a Scottish Home and Health Department Vacation Scholarship. W. H. McBride is indebted to the Cancer Research Campaign for grant support; M.F.A.W. and G.M.F. to the Medical Research Council for a Project Grant and to Professor H. J. Evans for the privilege of working in his unit.

### REFERENCES

ECCLES, S. A. (1978) Macrophages and Cancer. In *Immunological Aspects of Cancer* (Ed. Castro). Lancaster: MTP Press.

EVANS R. (1972) Macrophages in syngeneic animal tumours. *Transplantation*, **14**, 468.

GEBHARDT, M. C. & FISHER, B. (1979) Further observations on the inhibition of tumour growth by *Corynebacterium parvum*. IX. Macrophage content of tumours in mice. *J. Natl Cancer Inst.*, **62**, 1034.

JAMES, K., MCBRIDE, W. H. & STUART, A. (Eds) (1977) *The Macrophage and Cancer.* Edinburgh: Published by the editors.

MCBRIDE, W. H. & MOORE, K. (1982) The effect of *C. parvum* therapy on intratumoral macrophage subpopulations. In *Macrophage and NK Cell Regulation and Function* (Ed. Sorkin & Norman) (in press).

MOORE, K. & MCBRIDE, W. H. (1980) The activation state of macrophage subpopulations from a murine fibrosarcoma. *Int. J. Cancer*, **26**, 609.

PROSS, H. F. & KERBEL, R. S. (1976) An assessment of intratumor phagocytic and surface marker-bearing cells in a series of autochthonous and early passaged chemically-induced murine sarcomas. *J. Natl Cancer Inst.*, **57**, 1157.

THOMSON, A. W., CRUICKSHANK, N. & FOWLER, E. F. (1979) Fc receptor-bearing and phagocytic cells in syngeneic tumours of *C. parvum* and carrageenan-treated mice. *Br. J. Cancer*, **39**, 598.

WOODRUFF, M. F. A. (1980) *The Interaction of Cancer and Host: Its Therapeutic Significance.* New York: Grune and Stratton Inc.

WOODRUFF, M. F. A., FORBES, G. M. & GORDON, J. (1982*a*) Immunogenicity, macrophage sensitivity and therapeutic response to *C. parvum* of fibrosarcomas induced in *C. parvum*-treated and untreated mice. *Cancer Immunol. Immunoth.*, **12**, 255.

WOODRUFF, M. F. A., FORBES, G. M. & SPEEDY, G. (1982*b*) Further studies on the inhibition of chemical carcinogenesis by *C. parvum*. *Cancer Immunol. Immunoth.*, **12**, 259.